# Experimental analysis of oil flow and drag torque generation in disengaged wet clutches

**DOI:** 10.1038/s41598-023-43695-6

**Published:** 2023-10-11

**Authors:** Lukas Pointner-Gabriel, Elias Schermer, Thomas Schneider, Karsten Stahl

**Affiliations:** Department of Mechanical Engineering, School of Engineering and Design, Gear Research Center (FZG), Technical University of Munich, Boltzmannstrasse 15, 85748 Garching near Munich, Germany

**Keywords:** Mechanical engineering, Fluid dynamics

## Abstract

Fundamental knowledge of the oil flow in a disengaged wet clutch is essential for optimizing the cooling performance and the drag losses. However, no fundamental information on the oil flow and drag torque generation is available for dip-lubricated wet clutches. Therefore, the oil flow and drag torque generation in the sub-millimeter gap of a dip-lubricated wet clutch was experimentally investigated for three practically relevant oil levels. To enable optical access to the gap, transparent components were used. Further, a high-speed camera was used to capture the oil flow in the gap and grooving. Independent of the set oil level, the gap is oil-filled at low differential speeds, resulting in a single-phase flow. The drag torque increases approximately linearly with increasing differential speed due to the fluid shearing. In certain regions of the waffle grooving, air bubbles form locally. The air bubbles preferably occur in the grooves oriented in the radial direction, while the grooves oriented in the peripheral direction are filled with oil. Above a certain differential speed, the oil is continuously displaced from the gap, starting from the inside, due to the increasing centrifugal force. Consequently, the drag torque increases in a degressive manner until a maximum value is finally reached. The ongoing displacement of oil from the gap eventually results in a decrease in the drag torque. A steady drag torque is generated only when the oil is almost entirely displaced from the gap. Since the oil displacement from the gap already commences at a low differential speed, the cooling performance is limited for dip-lubricated wet clutches. The continuous displacement of oil from the gap can be held up, among other things, by increasing the oil level.

## Introduction

In wet-running multi-plate clutches (hereinafter referred to simply as wet clutches), hydrodynamic drag losses occur—in the disengaged state and under differential speed—mainly due to the shearing of the oil in the sub-millimeter gaps. These losses can considerably reduce the overall efficiency of the drivetrain^1^. Consequently, there is a permanent need to reduce the drag losses of wet clutches. Today’s research focuses primarily on optimizing design and operating conditions, as well as alternative shifting element concepts^2^. The drag losses of wet clutches mainly depend on the clutch geometry, operating conditions, oil properties, type of lubrication, and various other influencing parameters^3–8^. The type of lubrication is usually chosen depending on the application and the requirements. In the case of injection lubrication, the oil is continuously injected centrally from the inside into the gaps. In contrast, in the case of dip lubrication, the clutch components permanently dip in an oil sump. In general, dip lubrication requires lower technical effort to be implemented than injection lubrication. Both the feeding flow rate in the case of injection lubrication and the oil level in the case of dip lubrication are decisive parameters for adjusting the cooling performance. However, a high feeding flow rate and oil level lead to high drag losses^3,4^. Consequently, it is generally not possible to maximize the cooling performance and minimize the drag losses simultaneously. Fundamental knowledge of the flow behavior in the gaps is essential for optimizing the cooling performance and the drag losses. For injection lubrication, the flow behavior in a disengaged wet clutch has been extensively investigated through experiments^9–11^ and simulations^12–16^. However, to the best of our knowledge, no fundamental information is available on the oil flow and drag torque generation in the gaps of a dip-lubricated wet clutch.

### Drag loss behavior and flow behavior in the case of injection lubrication

**Figure 1:**
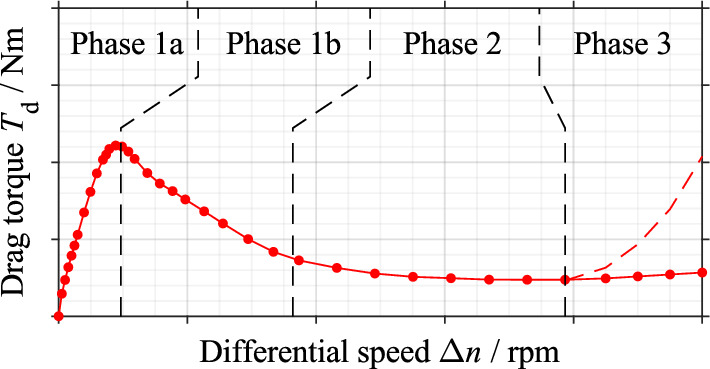
Characteristic drag torque curve when using injection lubrication or dip lubrication, as well as its classification^24^.

The subject of many research projects is investigating the drag loss behavior and oil flow in the gap when using injection lubrication. In essential studies, an understanding of the underlying physical mechanism of the flow development in the gap was gained^16^. Also, the influence of various parameters on the integral drag torque was investigated in many studies, both experimentally and by simulation^4,6,8,17,18^. Besides, many studies focused on investigating complex flow scenarios in the grooves of different groove designs and the local drag torque generation^10,12^. In the case of injection lubrication, the oil is continuously injected centrally from the inside into the gaps. The resulting flow pattern mainly depends on the acting centrifugal and viscous forces, as well as the surface tension^19^. The centrifugal force acting is comparatively small in the lower differential speed region. As a consequence, the radial flow velocity continuously decreases towards the outer radius according to the law of continuity^19^. Therefore, the gap is filled with oil, resulting in a single-phase flow. In this differential speed region, the drag torque increases approximately linearly (see Fig. 1, Phase 1a). Above a certain differential speed, the now dominant centrifugal force drives the oil outwards and hinders the deceleration of the radial flow^19^. As a result, air enters the gap, and a two-phase flow is formed^16,20^. The reduced viscosity of the oil-air mixture leads to a decrease in the drag torque (see Fig. 1, Phase 1b)^16,20^. The aeration area continues to grow as the differential speed is further increased until the gap is almost entirely filled with air^16^. The drag torque is almost steady at that point (see Fig. 1, Phase 2). In general, the flow behavior is decisively influenced by the operating parameters and the groove designs. Independent of the lubrication method, a re-increase in the drag torque may occur due to plate tumbling or plate movement at very high differential speeds (see Fig. 1, Phase 3)^21–23^.

### Drag loss behavior in the case of dip lubrication

In contrast to injection lubrication, the oil is not fed actively into the gaps in the case of dip lubrication. The drag loss behavior of dip-lubricated wet clutches was investigated only in a few studies^3,18^. Pointner-Gabriel et al.^3^ found in a preliminary study for the investigations presented in this publication that the characteristic behavior of the drag torque is comparable for injection lubrication and dip lubrication (see Fig. 1). However, it cannot be assumed that the flow behavior is comparable for both lubrication methods. Pointner-Gabriel et al.^3^ assume that the gaps are filled with oil in the range of low differential speeds. In this phase, the drag torque increases with respect to the differential speed (see Fig. 1, Phase 1a). As the differential speed increases, it is assumed that the oil is continuously displaced from the gaps. Consequently, the drag torque drops to a nearly steady plateau (see Fig. 1, Phase 1b). The generally low drag torque in Phase 2 (see Fig. 1) supports the hypothesis that oil is displaced from the gaps at high differential speeds. In addition, the influence of various parameters on drag loss behavior was investigated. It was found that the oil level influences the resulting drag torque, among other things.

### Oil flow visualization methods

Depending on the objective of the investigation, different systems can be applied to visualize the oil flow in the gaps of wet clutches. To capture the complex and small flow structures of the single-phase flow in the sub-millimeter gaps and in the grooves of the friction plates, the Defocusing Particle Tracking Velocimetry (DPTV)^10,25^ and the Laser Doppler Velocimetry Profile Sensor (LDV-PS)^26^ can be applied. Combining a planar Particle Image Velocimetry (PIV) system and a LDV-PS allows a large field of view to be covered in peripheral direction and the axial velocity profile to be captured in high resolution^27^. However, the methods mentioned above require transparent components to enable optical access to the gap. Using Dynamic Neutron Radiography (dNR), the flow visualization in the gaps of a wet clutch can be performed in an application-oriented test environment^28^. In order to reduce the attenuation of the neutron radiation, various test rig components need to be made of aluminum, as do the plates^28^. It can be summarized that the visualization methods mentioned are suitable for visualizing specific details in particular or determining velocity fields of the oil flow, even in the grooves of the friction plate, for example. However, the methods mentioned above are associated with high implementation effort and are complex in their application.

Alternatively, camera systems can be used to capture the oil flow. Using a camera system is usually sufficient to derive general statements about the flow behavior. Using a high-speed camera extends the scope to very short events, such as the formation and movement of air bubbles. Transparent components are necessary to obtain optical access to the gap. Due to the simple application, this method is applied in the vast majority of the studies^4,11,12,29–32^. Here, the frontmost plate of the clutch pack is usually replaced by a stationary acrylic glass pane. Consequently, the investigations are commonly performed in brake operating mode with the friction plate rotating. However, each of the methods mentioned above requires adaptations of the real system.

In addition, CFD (computational fluid dynamics) simulations can be performed to investigate the flow behavior in the gaps and grooves. Most of the studies focus mainly on the investigation of different groove geometries and the onset of aeration, as well as on the development of the two-phase flow^12–14,30,33–36^. The CFD models are usually validated based on integral drag torque measurements and high-speed recordings of the flow in the gap^12,29,36^.

### Objective

The overall objective of this study was the analysis and investigation of the general flow development and drag torque generation in the gap of a disengaged wet clutch when using dip lubrication. Further, the investigation of the oil level on the flow development was part of this study.

## Materials and methods

### Test set-up

**Figure 2:**
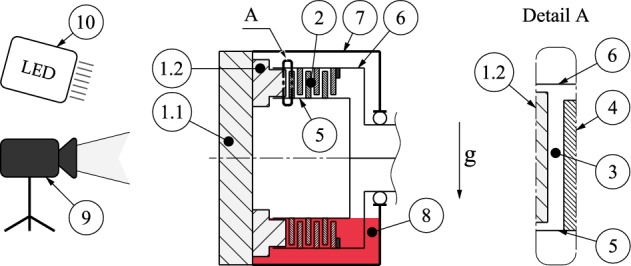
Side view of the schematic experimental set-up to investigate the flow in the frontmost gap of a dip-lubricated wet clutch.

Since this study aimed to investigate the general flow development in the gap, transparent components combined with a high-speed camera were used to visualize and capture the oil flow in the gap and grooves. The investigations were performed on the LK-4 drag torque test rig (Gear Research Center (FZG), Technical University of Munich). A detailed description of the test rig can be found in the publication of Pointner-Gabriel et al.^24^. The experimental set-up is shown schematically in Fig. 2. A full clutch pack was used to ensure practical relevance, although only the flow in the frontmost gap was considered. Thus, compared to investigations performed on single-plate test rigs, the potential influence of the reduced number of plates on flow development can be eliminated. The investigations are based on the widely applied waffle groove design. In most of the experimental studies on the oil flow in the gap, the comparably less complex radial groove design was used^4,10,12,29,30^. To reduce the complexity of the flow, many studies are based only on non-grooved plates ^11,31^.

**Figure 3:**
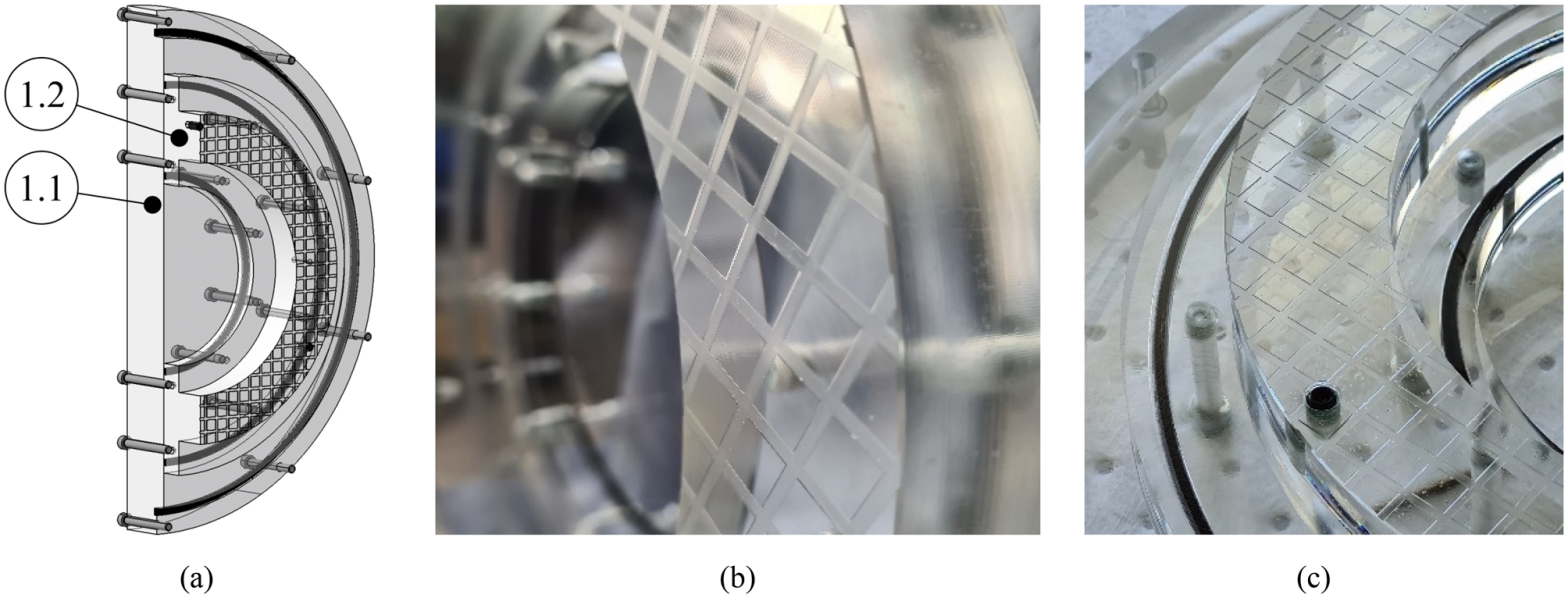
Different views of the transparent front cover, deployed to enable optical access to the flow in the gap: (**a**) graphical display of the front cover with ring and integrated waffle groove design; (**b**) detail of the front cover with waffle groove design and threaded pin to limit the clearance; (**c**) detail of the milled waffle groove design. Note: The front cover is sectioned in (**a**) for illustrative purposes.

To visualize the flow in the gap, the closing front cover (consisting of parts 1.1 and 1.2) consists of acrylic glass components. The front cover simultaneously represents the frontmost plate of the clutch pack (2) and consists of a planar pane (1.1) and a ring of acrylic glass with an integrated waffle groove design (1.2) (see Fig. 3a). Thus, the gap (3) is bounded axially by the front cover fixed to the housing and an inner plate (4), and radially by the inner carrier (5) and the outer carrier (6). The test rig housing (7) is filled with oil (8) up to a predefined level for the investigations. The high-speed camera (9) and LED spotlight (10) are placed in front of the test rig.

The transparent front cover with the integrated waffle groove design is shown in Fig. 3. The cross-section of the groove is rectangular, with a groove depth of 0.25 mm and a groove width of 2 mm. The grooves have a spacing of 10 mm in each of the two main directions and were manufactured by milling. Figure 3b shows a detailed view of the grooves. The grooving covers approximately 17% of the ring area. The groove volume is approximately 2150 mm^3^. The clearance of the considered gap is limited by three threaded pins in the acrylic glass ring (see Fig. 3c). The pins simultaneously prevent too small a clearance and the surface from being scratched due to contact with the rotating separator plate.

**Figure 4:**
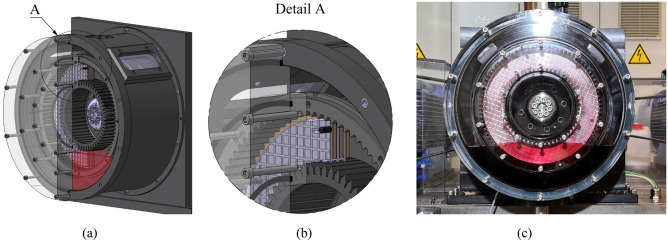
Different views of the front cover mounted on the housing of the test rig: (**a**) graphical display of the front cover mounted; (**b**) detail of the front cover with the threaded pin to limit the clearance; (**c**) front view of the test rig and set oil level. Note: The front cover is sectioned in (**a**) and (**b**) for illustrative purposes.

Figure 4 shows different views of the front cover mounted on the housing of the test rig. Since this investigation focused primarily on the visualization of the flow in the gap, no oil temperature control was used. Consequently, the oil temperature and, thus, the oil viscosity may vary slightly due to the continuous oil shearing during the tests. The influence of the temperature variation on the flow behavior is considered to be of minor relevance and was therefore accepted. In contrast to the test set-up used in the preliminary study^3^, the oil level was not controlled during the runs but set initially to a defined level, as in real applications.

A high-speed camera (Photron Fastcam Mini AX200) was used to capture the flow behavior. The camera delivers an image recording rate of 6400 fps at a maximum image resolution of 1024 × 1024 pixels. The camera’s lens was set to the maximum focal length of 75 mm for all shots to maximize the distance to the object and, thus, minimize the distortion. To realize the high image recording rate, the camera was combined with a LED spotlight (non-pulsed) with a maximum luminous flux of 36,000 lm.

### Clutch plates

**Figure 5:**
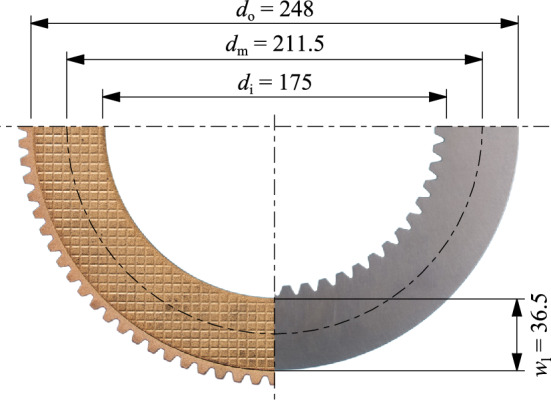
Dimensions (mm) of a friction plate (left; waffle groove design) and separator plate (right) of D212 clutch size.

In this study, clutch plates from the serial production of an industrial application, namely D212 (see Fig. 5) were used. The inner plate is designed as the separator plate, while the outer plate is the friction plate with a sinter friction lining and a waffle groove design. The clutch pack consists of three separator plates and three friction plates (see Fig. 2). The fourth and frontmost friction plate of the clutch pack is represented by the acrylic glass ring with the integrated groove design (see section “Test set-up”). The lining width *w*_l_ is 36.5 mm. The D212 clutch size was also part of the investigations in the preliminary study^3^.

### Oil

The tests were performed with an oil commonly used in the industrial setting. The physical characteristics of the oil are listed in Table 1. To improve the contrast, 2 ml of red dye was added. No influence on the results is expected from this.

**Table 1:** Physical characteristics of the oil used.

Density at 15 °C	878 kg/m^3^
Kinematic viscosity at 40 °C	93.3 mm^2^/s
Kinematic viscosity at 100 °C	14.4 mm^2^/s

### Test procedure and evaluation methodology

Due to its high practical relevance, the brake operating mode with a rotating inner carrier was investigated. In this case, the outer carrier is stationary. The tests were performed according to the test procedure presented in the publication of Pointner-Gabriel et al.^24^. Within the test runs, the inner carrier was first accelerated to a starting speed of 800 rpm, followed by a stepwise reduction of the speed to zero. Compared to a constant speed ramp, influences arising from transient effects due to a speed change can be avoided thanks to the stepwise nature of the speed change. It is known from prior tests that Phase 2 is already reached at the chosen starting speed. After mounting the plates, the set total clearance is generally not evenly distributed among the single gaps. To reduce the influence of the uneven distribution of the total clearance on the drag torque^37^, the starting speed was held for at least 120 s. This results in a more even distribution of the set total clearance. Further, it was observed through an inspection window in the housing that the plate positions do not significantly vary within the tests. The step size and step duration of the inner carrier speed were set depending on the test phase, and usually ranged from 25 to 100 rpm and 60–90 s, respectively. The oil flow was recorded at the end of each speed step. In this publication, representative images are used for discussing the flow behavior. The high-speed video recordings are provided as supplementary material. To prove the reliability of the results, the video recordings were systematically repeated.

The drag loss behavior and flow development at increasing speed steps was determined in a screening test. There was no systematic deviation found between increasing and decreasing speed steps.

The drag torque measurements were evaluated according to the evaluation methodology presented in the publication of Pointner-Gabriel et al.^24^. Here, the mean drag torque is determined for each differential speed step from the measured drag torque curve. Unsteady effects caused by deceleration are eliminated by skipping the first 70% of the drag torque signal in each step. Then, the arithmetic means of the last 30% of the continuous drag torque signal for each step of a constant differential speed are calculated. The averaging allows the determination of the steady values. The onset of Phase 2 was also determined according to the methodology of Pointner-Gabriel et al.^24^.

The measurement uncertainty of the drag torque was determined with the standard GUM (Guide to the expression of uncertainty in measurements) method described in ISO/IEC GUIDE 98-3:2008^38^. The method is based on model equations, which represent the measurement chain and contain quantities that contribute to the measurement uncertainty. A confidence level of *p* = 95.45% was chosen, as it is commonly used for reporting measurement uncertainty. The expected measurement uncertainty of the drag torque is shown in Supplementary Fig. 1. The oil level was evaluated based on the pixels of the images. The resolution of the oil level value is approximately 0.4 mm/px. The oil level in the zone where the plates exit the oil sump (see yellow mark on the right in Fig. 8) was always slightly higher than in the zone where the plates enter the oil sump (see yellow mark on the left in Fig. 8). Thus, the oil levels of both zones were averaged to evaluate the oil level variation. The non-linear change in oil level due to the circular cross-section of the housing is neglected. In addition to the high-speed recordings, the flow was observed visually to generate further knowledge.

### Test conditions

**Figure 6:**
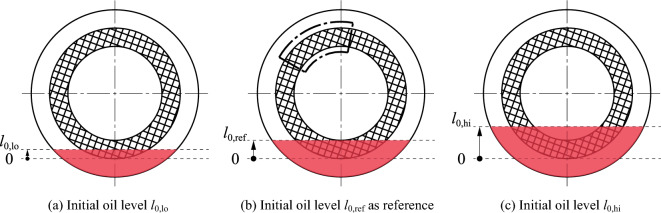
Schematic representation of the initial oil levels investigated and field of view for close-up recordings. Note: lo, low; ref, reference; hi, high.

A nominal clearance of 0.35 mm was set per gap. The direction of rotation was positive (counterclockwise) in all tests. The possible influence of the direction of rotation on the flow behavior was investigated in preliminary tests and can be excluded. At the beginning of the test, the oil temperature was approximately 25 °C. The oil level is defined by the immersion depth of the plates and is given as a proportion of the lining width *w*_l_. The initial oil levels investigated are shown schematically in Fig. 6.

- Initial oil level *l*_0,lo_ = 0.5 × *w*_l_ = 18.3 mm, see Fig. 6a.
- Initial oil level *l*_0,ref_ = *w*_l_ = 36.5 mm, see Fig. 6b; used as reference.
- Initial oil level *l*_0,hi_ = 1.75 × *w*_l_ = 63.9 mm, see Fig. 6c.

At the initial oil level *l*_0,hi_ the inner carrier dips into the oil sump.

## Results

In order to improve the understanding of the acting phenomena, as an introduction, the relation of the oil flow and the drag torque for the initial oil level *l*_0,ref_ is described in detail. Based on this, the results for the investigated initial oil levels *l*_0,lo_ and *l*_0,hi_ are shown. Although a stepwise reduction of the differential speed is performed when running the test, the results are interpreted and discussed using the established representation of drag torque behavior with respect to increasing differential speed. In order to evaluate the flow in the grooves, additional close-up video recordings were taken (field of view see Fig. 6b). The oil-filled gap and the oil-filled grooves can be distinguished by their color intensity. Due to the locally wider distance, the oil-filled grooves appear darker (see Fig. 9). Air bubbles are visible as light areas (see Fig. 9).

### Relation of flow pattern and drag torque

**Figure 7:**
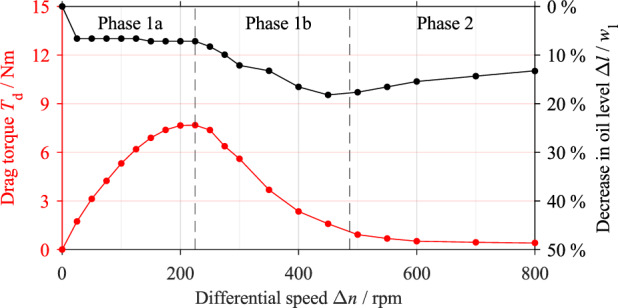
Drag torque and decrease in oil level with respect to the differential speed for the initial oil level *l*_0,ref_.

Figure 7 shows the drag torque curve for the initial oil level *l*_0,ref_ and its classification into characteristic phases, as well as the decrease in oil level. In Phase 1a, the drag torque *T*_d_ increases with respect to the differential speed Δ*n* and reaches a maximum value of 7.675 Nm ±﻿ 0.074 Nm at Δ*n* = 225 rpm. The oil level drops abruptly by approximately 7% at the onset of rotation but is nearly constant for the rest of Phase 1a. In Phase 1b, the drag torque decreases continuously and reaches a steady value at approximately Δ*n* = 485 rpm. This marks the onset of Phase 2. In Phase 1b, the oil level first decreases continuously by 18% to a minimum level before increasing again from Δ*n* = 450 rpm. In Phase 2, the slight increase in the oil level continues. At the maximum differential speed of Δ*n* = 800 rpm, the oil level is about 13% lower than the initial oil level.

**Figure 8:**
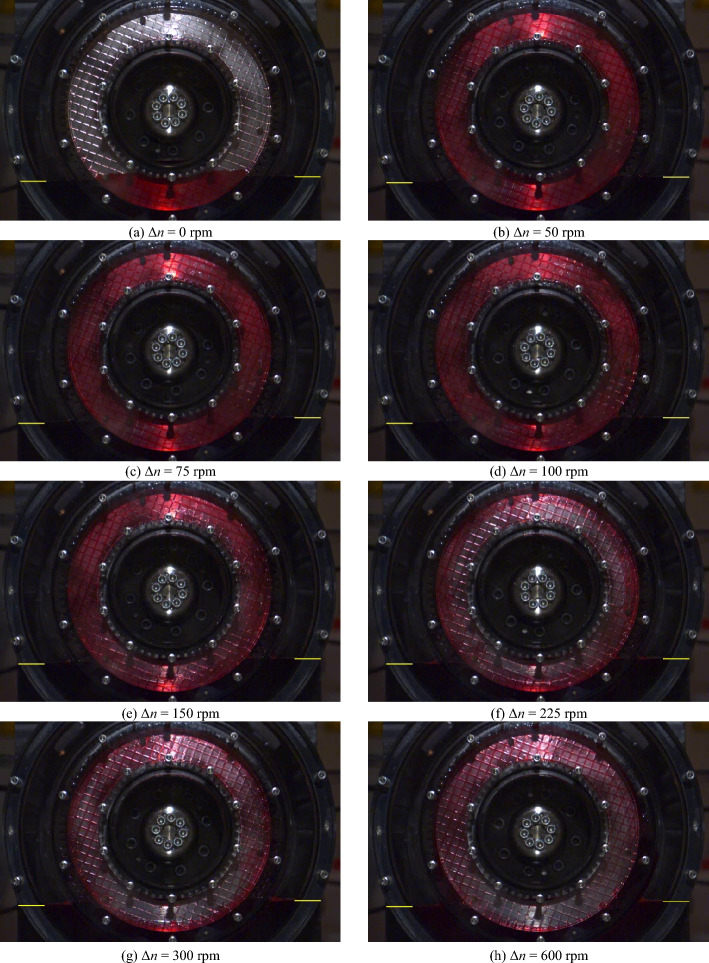
Flow patterns at specific differential speeds for the initial oil level *l*_0,ref_. Note: For the high-speed video recordings, see Supplementary Video 1.

**Figure 9:**
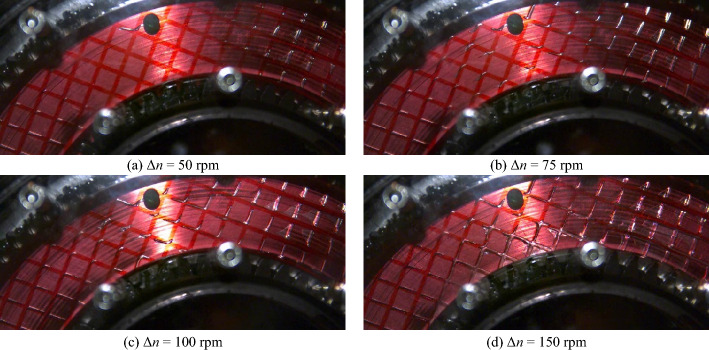
Close-up images of the flow at specific differential speeds for the initial oil level *l*_0,ref_. Note: For the high-speed video recordings, see Supplementary Video 2.

Figure 8 shows the flow patterns in the gap for specific differential speeds for the initial oil level *l*_0,ref_. The corresponding high-speed video recordings are shown in Supplementary Video 1. A selection of close-up images of the flow is shown in Fig. 9. The corresponding close-up high-speed video recordings are shown in Supplementary Video 2.

Figure 8a shows the partially filled gap between the front cover and the separator plate at zero speed. The capillary effect causes the oil in the narrow gap to be slightly higher than in the surrounding oil sump. At Δ*n* = 50 rpm, the gap is filled with oil (see Fig. 8b), while air bubbles appear in certain regions of the grooving (see Fig. 9a). As the close-up shows, the air bubbles tend to form where the grooves are oriented in or nearly in the radial direction. In contrast, the grooves are oil-filled if they are oriented in or nearly in the peripheral direction. As a consequence of the increase in differential speed to Δ*n* = 75 rpm or Δ*n* = 100 rpm, respectively, the air bubbles continue to spread (see Fig. 9b and c). Apart from this, the gap is still filled with oil from the inner to the outer diameter in this differential speed region (see Fig. 8b and c). At Δ*n* = 150 rpm, the increased conveying capacity of the clutch leads, in addition to the air bubbles (see Fig. 9d), to an oil-free area in the inner part of the gap (see Fig. 8e). The air bubbles partially spread across the grooves into the gap at this differential speed. As the differential speed increases, the oil-filled part continuously shrinks due to the increasing centrifugal force. At Δ*n* = 225 rpm and thus at the differential speed of the maximum drag torque, the gap is only filled with oil in the outer part (see Fig. 8f). At Δ*n* = 300 rpm, the displacement of the oil from the gap is advanced (see Fig. 8g). At Δ*n* = 600 rpm, the flow pattern is almost steady (see Fig. 8h). However, the conveying capacity of the clutch is not sufficient to entirely displace the oil from the gap, even in the section of the sump. The flow pattern remains virtually unchanged with a further increase in the differential speed. It was observed that oil accumulates inside the outer carrier depending on the differential speed. The oil partially runs back into the sump on the outside of the outer carrier. In a control test, similar drag loss behavior and flow development was observed for a non-grooved front cover.

### Influence of initial oil level on oil flow and drag loss behavior

#### Low oil level

**Figure 10:**
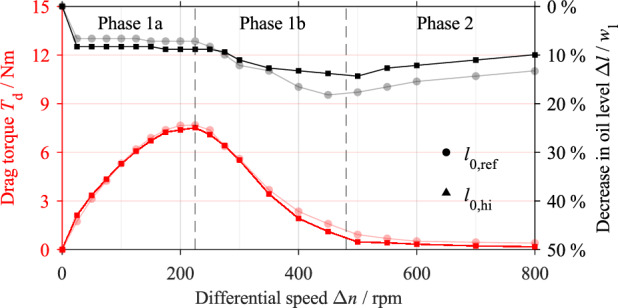
Drag torque and decrease in oil level with respect to the differential speed for the initial oil level *l*_0,lo_ in comparison to the reference test (light colors). Note: The classification of the drag torque curve refers to the initial oil level *l*_0,lo._

Figure 10 shows the drag torque curve and the decrease in the oil level for the initial oil level *l*_0,lo_ in comparison to the reference test results. Both the drag torque and oil level show similar behavior. The lower initial oil level results in a maximum drag torque of 7.519 Nm ± 0.072 Nm. Phase 2 begins at a differential speed of approximately Δ*n* = 480 rpm. At the onset of rotation, the oil level drops abruptly by approximately 8%. The oil level remains almost constant for the rest of Phase 1a. In Phase 1b, the oil level decreases continuously by 14% to a minimum level before increasing again in Phase 2. At the maximum differential speed of Δ*n* = 800 rpm, the oil level is about 10% lower than the initial oil level.

**Figure 11:**
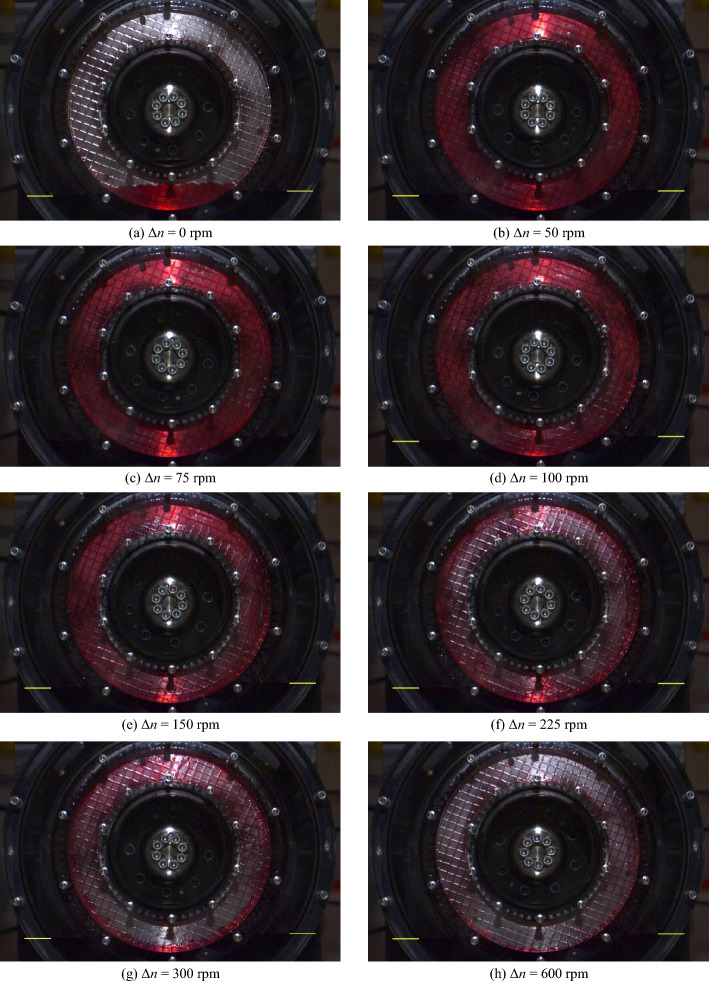
Flow patterns at specific differential speeds for the initial oil level *l*_0,lo_. Note: For the high-speed video recordings, see Supplementary Video 3.

Not only do the drag torque and the oil level change similarly to the reference test, but the oil flow does likewise. Figure 11 shows the flow patterns in the gap for specific differential speeds for the initial oil level *l*_0,lo_. The corresponding high-speed video recordings are shown in Supplementary Video 3. Figure 11a shows the partially filled gap between the front cover and the separator plate at zero speed. Up to a differential speed of approximately Δ*n* = 100 rpm, the gap is filled with oil (see Fig. 11b–d). In certain regions of the grooving, air bubbles can be observed. As the differential speed increases, a ring free of oil forms in the inner part at about Δ*n* = 150 rpm (see Fig. 11e). Compared to the reference test, the oil displacement from the gap is slightly more advanced in this differential speed region. As the differential speed increases, the oil-filled part continuously shrinks due to the increasing centrifugal force. At Δ*n* = 225 rpm and thus at the differential speed of the maximum drag torque, the gap is only filled with oil in the outer part (see Fig. 11f). At Δ*n* = 300 rpm, the oil displacement from the gap is advanced (see Fig. 11g). At Δ*n* = 600 rpm, the gap is almost free of oil (see Fig. 11h).

#### High oil level

**Figure 12:**
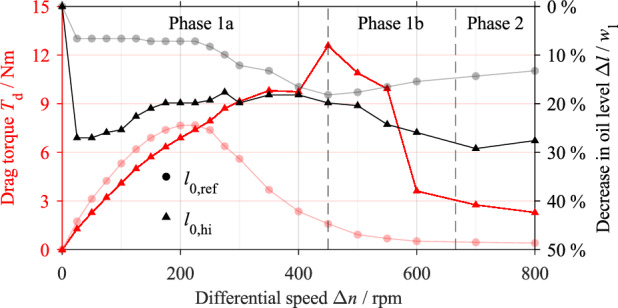
Drag torque and decrease in oil level with respect to the differential speed for the initial oil level *l*_0,hi_ in comparison to the reference test (light colors). Note: The classification of the drag torque curve refers to the initial oil level *l*_0,hi._

Figure 12 shows the drag torque behavior and the decrease in the oil level for the initial oil level *l*_0,hi_ in comparison to the reference test results. The maximum drag torque is reached at Δ*n* = 450 rpm and is 12.571 Nm ± 0.120 Nm. The characteristic drop of the drag torque in Phase 1b ends at approximately Δ*n* = 665 rpm. At the onset of rotation, the oil level drops sharply because the inner carrier transports oil out of the sump. The oil level rises first as the differential speed increases, then drops again after the maximum drag torque is passed.

**Figure 13:**
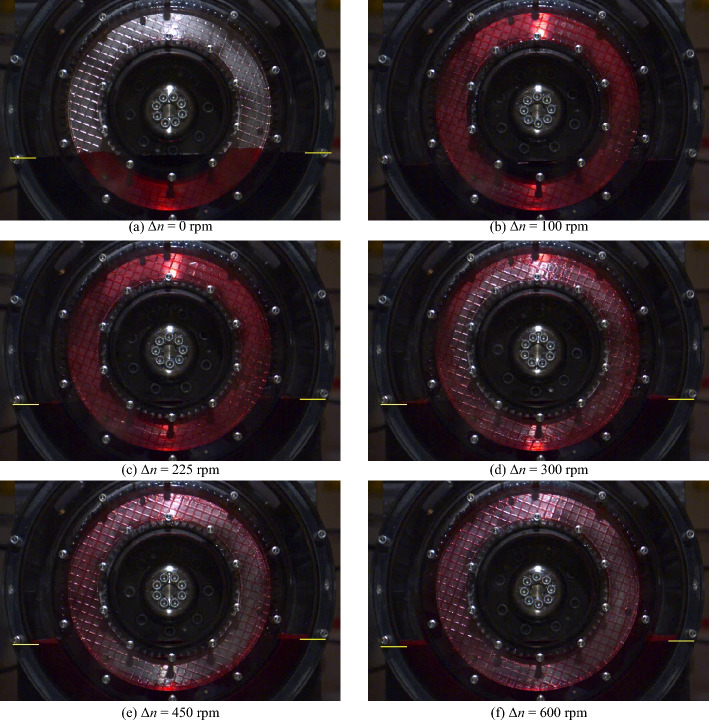
Flow patterns at specific differential speeds for the initial oil level *l*_0,hi_. Note: For the high-speed video recordings, see Supplementary Video 4.

Figure 13 shows the flow patterns in the gap for specific differential speeds for the initial oil level *l*_0,hi_. The corresponding high-speed video recordings are shown in Supplementary Video 4. The flow in the gap develops similarly to the reference test even at the high oil level. Figure 13a shows the partially filled gap between the front cover and the separator plate at zero speed. Up to a differential speed of approximately Δ*n* = 225 rpm, the gap is filled with oil (see Fig. 13b and c). Beginning at Δ*n* = 250 rpm, and thus significantly later than in the reference test, an oil-free area forms in the inner region even at the initial oil level *l*_0,hi_. Figure 13d shows the flow pattern at Δ*n* = 300 rpm. At Δ*n* = 450 rpm and thus at the differential speed of the maximum drag torque, oil only enters the gap in the section of the sump (see Fig. 13e). At Δ*n* = 600 rpm, the gap is almost free of oil (see Fig. 13f).

## Discussion

### Flow development and drag torque generation

**Figure 14:**
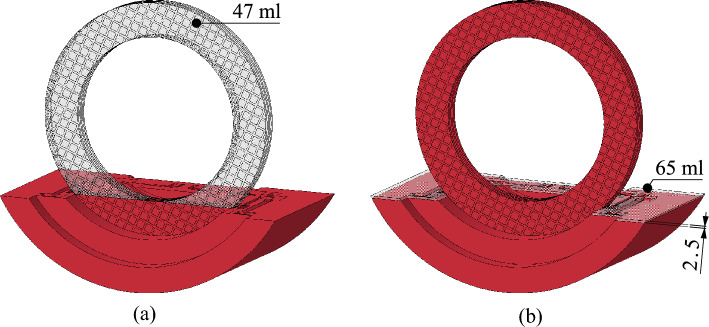
Simplified CAD model of the flow domain consisting of the oil sump and six gaps for the initial oil level *l*_0,ref_: (**a**) Oil distribution at zero speed; (**b**) simplified oil distribution at low differential speed in Phase 1a. Note: Dimensions in mm.

The flow development and drag torque generation are discussed based on the reference test. The drag torque curve in Fig. 7 shows the characteristic phases identified in the preliminary study^3^. As the recordings (see Fig. 8) further show, the gap is filled with oil in the range of low differential speeds. The oil level in the housing drops accordingly. According to the CAD (computer aided design) model of the flow domain, the initial decrease in the oil level of about 7% (see Fig. 7), i.e. 2.5 mm, represents a volume of approximately 65 ml (see Fig. 14b). In contrast, the oil-free regions of the gaps and grooves at zero speed represent a volume of approximately 47 ml (see Fig. 14a). Thus, about 72% of the oil being transported is in the gaps. According to the observations, the rest of the oil mainly remains within the outer carrier. In general, the drag torque results mainly from the shearing of the oil in the gaps and from the acceleration of the oil. It can be concluded that the oil shearing in the gap is the main cause of the drag torque generation at low differential speeds.

The approximately linear increase in the drag torque at the beginning of Phase 1a can be explained using Newton's law of viscosity, which states that the drag torque increases linearly with the differential speed in the case of a filled gap. Due to the steady flow state, the oil level is nearly constant in this differential speed range. The continuous displacement of oil from the gap is related to the degressive increase in the drag torque towards the end of Phase 1a. The subsequent decrease in the drag torque is associated with the advanced displacement of the oil from the gap. As the differential speed increases, the oil is increasingly transported out of the sump, leading to a decrease in oil level at the beginning of Phase 1b, although the gaps are almost free of oil at this point. The re-increase in the oil level in Phase 1b and Phase 2 cannot be explained based on the flow patterns. One possible explanation for this behavior is that the high centrifugal force and the high rotational speed prevent the oil from adhering to the plates and, thus, from being transported beyond a certain speed. As a result, the oil level increases again. The steady drag torque in Phase 2 is reached when a steady flow state is reached. The results largely confirm the explanatory approaches and hypotheses stated in the preliminary study^3^ on the generation of drag losses. In contrast to injection lubrication, the oil-free area develops from the inside. Nevertheless, both lubrication methods lead to a comparable drag loss behavior.

### Influence of initial oil level on drag torque and flow patterns

The influence of the initial oil level on the drag torque and flow patterns is discussed compared to the reference test. The flow in the gap develops similarly in the case of the investigated oil levels (see Figs. 8, 11 and 13). However, the initial oil level defines the transition between the phases and the drag torque level. The drag torque values and uncertainties are listed in Supplementary Table 1 for comparison of the measurements. The low measurement uncertainties allow to show significant differences between the investigated oil levels.

#### Low oil level

The flow develops nearly identically for the low and the reference oil level (see Figs. 8 and 11). Figure 10 shows that the lower oil level results in a slightly lower drag torque. Moreover, Phase 2 is already reached at a slightly lower differential speed. Thus, the influence of the initial oil level on the drag torque behavior is consistent with the results of the preliminary study^3^. However, the lower oil level leads to only a minor drag torque decrease. Compared to the reference test, the oil level decreases slightly more in Phase 1a. According to the CAD model (analogous to Fig. 14) of the flow domain, the initial decrease in the oil level of about 8% (see Fig. 10), i.e. 3 mm, represents a volume of approximately 84 ml. Due to the lower initial oil level, the oil-free regions of the gaps and grooves at zero speed represent a comparably larger volume of approximately 58 ml. For this reason, the oil level drops to a lower level in Phase 1a compared to the reference test.

#### High oil level

At the high oil level, the immersing inner carrier causes additional drag losses. Due to the higher oil level, a higher centrifugal force is required to displace the oil from the gap. Accordingly, the oil-free area in the inner part of the gap begins to develop at a higher differential speed. Therefore, the maximum drag torque and the steady flow state are reached at comparably higher differential speeds. At the differential speed of the maximum drag torque, the gap is almost free of oil. Therefore, the drag torque in this phase is mainly caused by the conveying of the oil. At the high initial oil level, the conveying capacity of the clutch in the differential speed range of Phase 2 is still sufficient to displace the oil almost entirely from the gap. It is mainly the immersion of the inner carrier that dominates oil transportation at the high oil level. Therefore, the decrease in the oil level is not compared in detail. As a result of the overall higher power loss, the oil temperature increases continuously. This, in turn, reduces the oil viscosity and, thus, lowers the drag torque. The generally higher temperature level (see Supplementary Fig. 2) causes a slightly lower drag torque at the beginning of Phase 1a. The rotation of the clutch components causes a circulation in the sump. It is assumed that not only the speed-dependent energy input, but also the varying flow conditions in the sump cause the change in the temperature.

### Formation and movement of air bubbles

**Figure 15:**
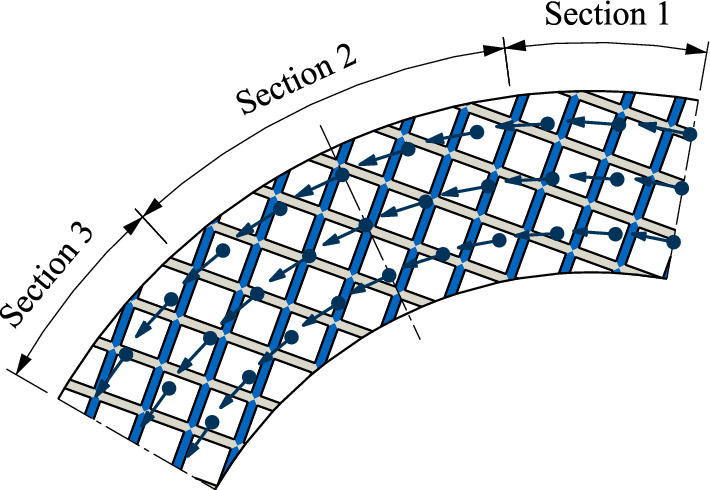
Schematic representation of the waffle groove design and simplified peripheral velocity field of the flow in the gap for the chosen field of view.

In contrast to injection lubrication, the oil is not actively fed into the gaps in the case of dip lubrication. In the case of dip lubrication, the flow in the gap is caused solely by the rotation of the plates. Therefore, the flow in the peripheral direction is assumed to be dominant under constant operating conditions. In order to derive explanations for the formation and movement of the air bubbles, the flow field’s axial and radial velocity components are neglected. The simplified velocity field of the flow in the filled gap is shown in Fig. 15. Further, Fig. 15 schematically represents the field of view covered by the close-up images. The orientation of the groove pattern in Fig. 15 is identical to the test set-up. In the schematic representation, the grooves are highlighted in gray or blue, depending on the orientation. Moreover, the section is divided into three sub-sections. In Section 1, the grooves highlighted in gray tend to be oriented in a peripheral direction, while those highlighted in blue tend to be oriented in a radial direction. In Section 2, the grooves highlighted in gray and blue tend to be diagonal to the peripheral and radial directions, respectively. In Section 3, the grooves highlighted in blue tend to be oriented in a peripheral direction, while those highlighted in gray tend to be oriented in a radial direction.

In the case of injection lubrication, cavitation was identified as reason for air bubble formation^31,39^. However, the air bubbles appeared at significantly higher differential speeds (Δ*n* = 400 rpm)^31^. Thus, it is assumed that in the case of dip lubrication the air bubbles are not caused by cavitation but represent enclosed air. For totally excluding cavitation as reason for the air bubble formation, further experimental or simulative investigations need to be performed. As the close-up images show, the air bubbles preferably form in the grooves oriented in or nearly in the radial direction in Section 1 and Section 3, while the grooves oriented in the peripheral direction are filled with oil (see Fig. 9a–d). Due to the geometric characteristics of the waffle grooving, the air de facto cannot escape from the groove segments oriented in the radial direction. In the transition zones between the sections, the air bubbles do not cover the whole segment of the groove. It can be assumed that the air bubbles move along the oil flow in the grooves. Thus, at the transition between Section 1 and Section 2, the air bubbles are mainly present in the inner parts of the groove segments due to the inwards orientated groove. In contrast, the air bubbles are mainly present in the outer parts of the groove segments at the transition between Section 2 and Section 3 due to the outwards orientated groove. In Section 2, the comparably smaller air bubbles form at the intersections of the grooves (see Fig. 9b and c). The high-speed video recordings show that smaller bubbles detach from the air bubbles and move through the grooves in the direction of the rotation. It is assumed that the air bubbles accumulate at the intersections due to the local deceleration of the flow. Supplementary Video 5 shows the start phase of flow development in the case of a constant acceleration of 25 rpm/s. Based on the flow behavior it can be assumed that the air bubbles are fed from the inside as a consequence of the conveying effect of the clutch. The formation and movement of air bubbles were also investigated with CFD simulations^12^.

## Limitations and outlook

The frontmost friction plate was replaced by a transparent ring of acrylic glass. It is assumed that using acrylic glass does not affect the flow behavior, although the plate material influences the drag torque in terms of the contact angle^20,40^. Further, the test set-up only allows investigations in brake operating mode. Investigations in clutch operating mode are not possible because of the rigid connection of the transparent ring to the housing. Due to the plate configuration used, with the separator plates as inner plates and the friction plates as outer plates, the investigations are based on a non-rotating grooving. Since the plates were not fixed by separating springs, for example, the clearance of the considered gap was allowed vary and deviate from the set nominal clearance. Due to the chosen set-up, the flow only in the frontmost gap of the clutch was visualized. It is assumed that the flow behavior in the other gaps develops similar. Furthermore, the results are based to the widely used waffle groove design. It is assumed that the formation of air bubbles, in particular, strongly depends on the groove design. The subject of follow-up investigations may be clutch size, oil viscosity, and clearance variations. However, in the case of dip lubrication, the oil will always be continuously displaced from the gap as described, irrespective of the groove design and the operating parameters set. In further studies, the CFD simulation may be used to analyze the unresolved effects in detail. Moreover, the CFD simulations can be performed without the limitations. The results of the present work can be used to validate the CFD model. Further, powerful visualization methods could be applied to capture the small flow structures in the gaps and in the grooves of the friction plates.

## Conclusions

The flow behavior in the gap of a dip-lubricated wet clutch was investigated experimentally for three representative oil levels. The test set-up represents a simple and practical way to investigate the flow behavior in a disengaged wet clutch. In the experimental investigations, the flow behavior in the sub-millimeter gap and the grooves was determined with respect to the differential speed. In contrast to injection lubrication, the oil-free area develops from the inside and grows with increasing differential speed. Nevertheless, both lubrication methods lead to a comparable drag loss behavior. When increasing the initial oil level, the onset of Phase 1b or Phase 2 occurs at a higher differential speed. Since the displacement of the oil from the gap starts even at low differential speeds, the cooling performance is limited when using dip lubrication. The oil displacement can be held up, among other things, by increasing the oil level.

### Supplementary Information

Supplementary Information 1.

Supplementary Video 1.

Supplementary Video 2.

Supplementary Video 3.

Supplementary Video 4.

Supplementary Video 5.

## Data Availability

The datasets generated or analyzed during the current study are available from the corresponding author on reasonable request.

## List of symbols

*d* (mm): Diameter
*l* (mm): Oil level
*p* (%): Confidence level
*P*_d_ (W): Power loss
*T*_d_ (Nm): Drag torque
*w*_l_ (mm): Lining width
Δ*l* (mm): Decrease in oil level
Δ*n* (rpm): Differential speed

## Indices

hi: High
i: Inner
lo: Low
m: Mean
o: Outer
ref: Reference
