# Supplementary material for: Experimental analysis of oil flow and drag torque generation in disengaged wet clutches
Source: Sci Rep. 2023 Oct 11;13:17193. doi: 10.1038/s41598-023-43695-6 (PMC10567852; doi:10.1038/s41598-023-43695-6)
Supplement: Supplementary file 1 — Supplementary Information 1. [file 41598_2023_43695_MOESM1_ESM.docx]

*Scientific Reports – Supplementary Information*

**Experimental analysis of oil flow and drag torque generation in disengaged wet clutches**

Lukas Pointner-Gabriel, Elias Schermer, Thomas Schneider, Karsten Stahl

Technical University of Munich, School of Engineering and Design, Department of Mechanical Engineering, Gear Research Center (FZG), Boltzmannstrasse 15, 85748 Garching near Munich, Germany

**Measurement uncertainty**


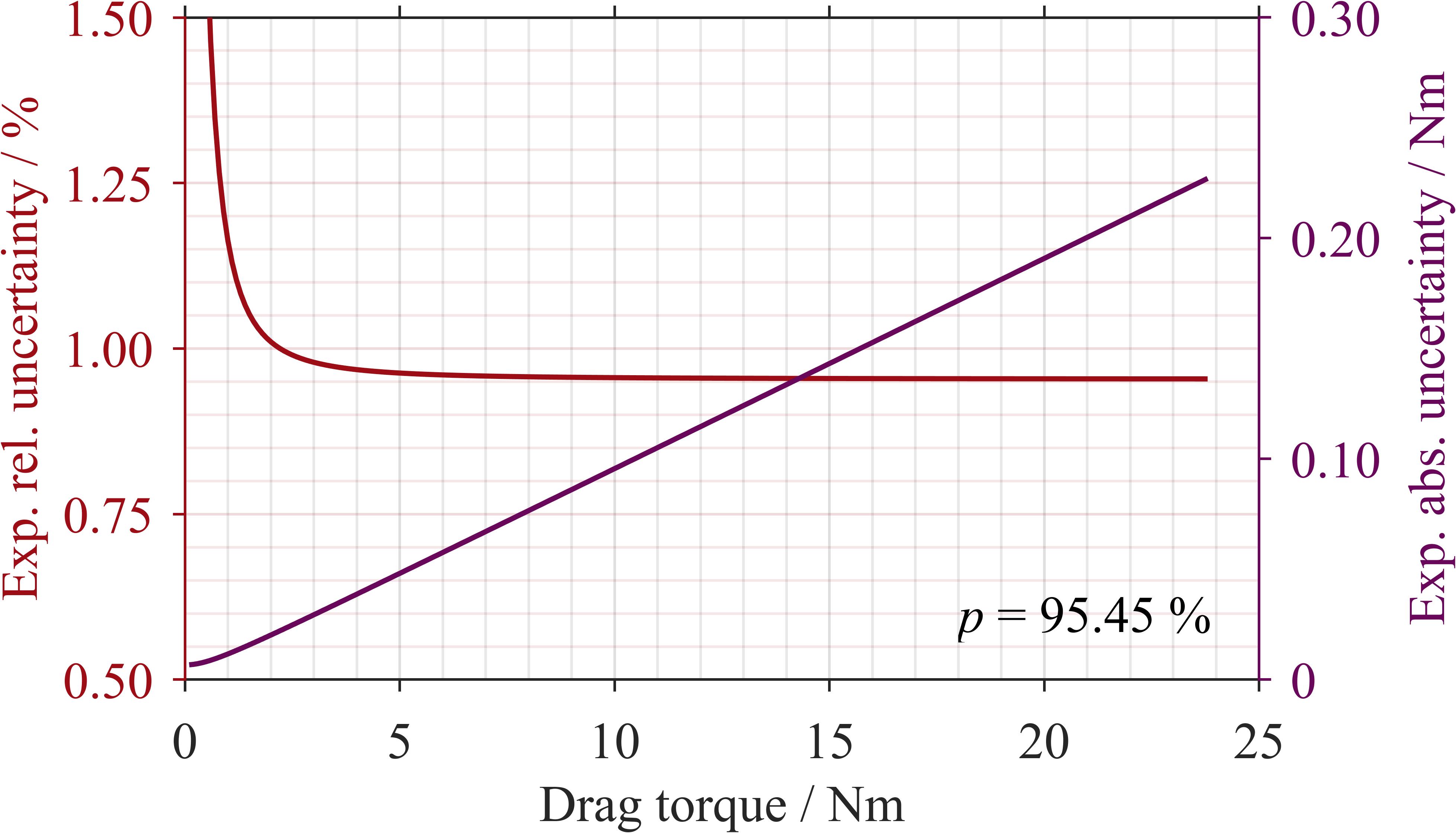


Supplementary Figure 1: Expected relative and absolute measurement uncertainty of the drag torque.

**Drag torque values and uncertainties**

Supplementary Table 1: Drag torque values and uncertainties (*p* = 95.45 %).

|  |  |  | Initial oil level *l*_0,lo_ | | |  |  | Initial oil level *l*_0,ref_ | | |  | |  | Initial oil level *l*_0,hi_ | | |
| --- | --- | --- | --- | --- | --- | --- | --- | --- | --- | --- | --- | --- | --- | --- | --- | --- |
| Δ*n* / rpm |  |  | *T*_d_ / Nm | | |  |  | *T*_d_ / Nm | | |  | |  | *T*_d_ / Nm | | |
| 0 |  |  | 0 |  |  |  |  | 0 |  |  |  | |  | 0 |  |  |
| 25 |  |  | 2.115 | ± | 0.021 |  |  | 1.741 | ± | 0.018 |  |  | 1.274 | ± | 0.014 |  |
| 50 |  |  | 3.350 | ± | 0.033 |  |  | 3.131 | ± | 0.031 |  |  | 2.280 | ± | 0.023 |  |
| 75 |  |  | 4.348 | ± | 0.042 |  |  | 4.241 | ± | 0.041 |  |  | 3.214 | ± | 0.031 |  |
| 100 |  |  | 5.293 | ± | 0.051 |  |  | 5.316 | ± | 0.051 |  |  | 4.100 | ± | 0.040 |  |
| 125 |  |  | 6.071 | ± | 0.058 |  |  | 6.194 | ± | 0.059 |  |  | 4.991 | ± | 0.048 |  |
| 150 |  |  | 6.711 | ± | 0.064 |  |  | 6.894 | ± | 0.066 |  |  | 5.713 | ± | 0.055 |  |
| 175 |  |  | 7.239 | ± | 0.069 |  |  | 7.382 | ± | 0.071 |  |  | 6.331 | ± | 0.061 |  |
| 200 |  |  | 7.381 | ± | 0.071 |  |  | 7.656 | ± | 0.073 |  |  | 6.889 | ± | 0.066 |  |
| 225 |  |  | 7.519 | ± | 0.072 |  |  | 7.675 | ± | 0.074 |  |  | 7.397 | ± | 0.071 |  |
| 250 |  |  | 7.088 | ± | 0.068 |  |  | 7.375 | ± | 0.071 |  |  | 7.931 | ± | 0.076 |  |
| 275 |  |  | 6.415 | ± | 0.052 |  |  | 6.376 | ± | 0.061 |  |  | 8.708 | ± | 0.083 |  |
| 300 |  |  | 5.527 | ± | 0.053 |  |  | 5.602 | ± | 0.054 |  |  | 9.136 | ± | 0.087 |  |
| 350 |  |  | 3.429 | ± | 0.033 |  |  | 3.688 | ± | 0.036 |  |  | 9.807 | ± | 0.094 |  |
| 400 |  |  | 1.926 | ± | 0.020 |  |  | 2.356 | ± | 0.023 |  |  | 9.730 | ± | 0.093 |  |
| 450 |  |  | 1.115 | ± | 0.013 |  |  | 1.594 | ± | 0.017 |  |  | 12.571 | ± | 0.120 |  |
| 500 |  |  | 0.474 | ± | 0.008 |  |  | 0.927 | ± | 0.011 |  |  | 10.893 | ± | 0.104 |  |
| 550 |  |  | 0.423 | ± | 0.008 |  |  | 0.688 | ± | 0.009 |  |  | 9.916 | ± | 0.095 |  |
| 600 |  |  | 0.335 | ± | 0.007 |  |  | 0.521 | ± | 0.008 |  |  | 3.611 | ± | 0.035 |  |
| 700 |  |  | 0.228 | ± | 0.007 |  |  | 0.452 | ± | 0.008 |  |  | 2.758 | ± | 0.027 |  |
| 800 |  |  | 0.176 | ± | 0.007 |  |  | 0.407 | ± | 0.008 |  |  | 2.292 | ± | 0.023 |  |

**Power loss and change in sump temperature**


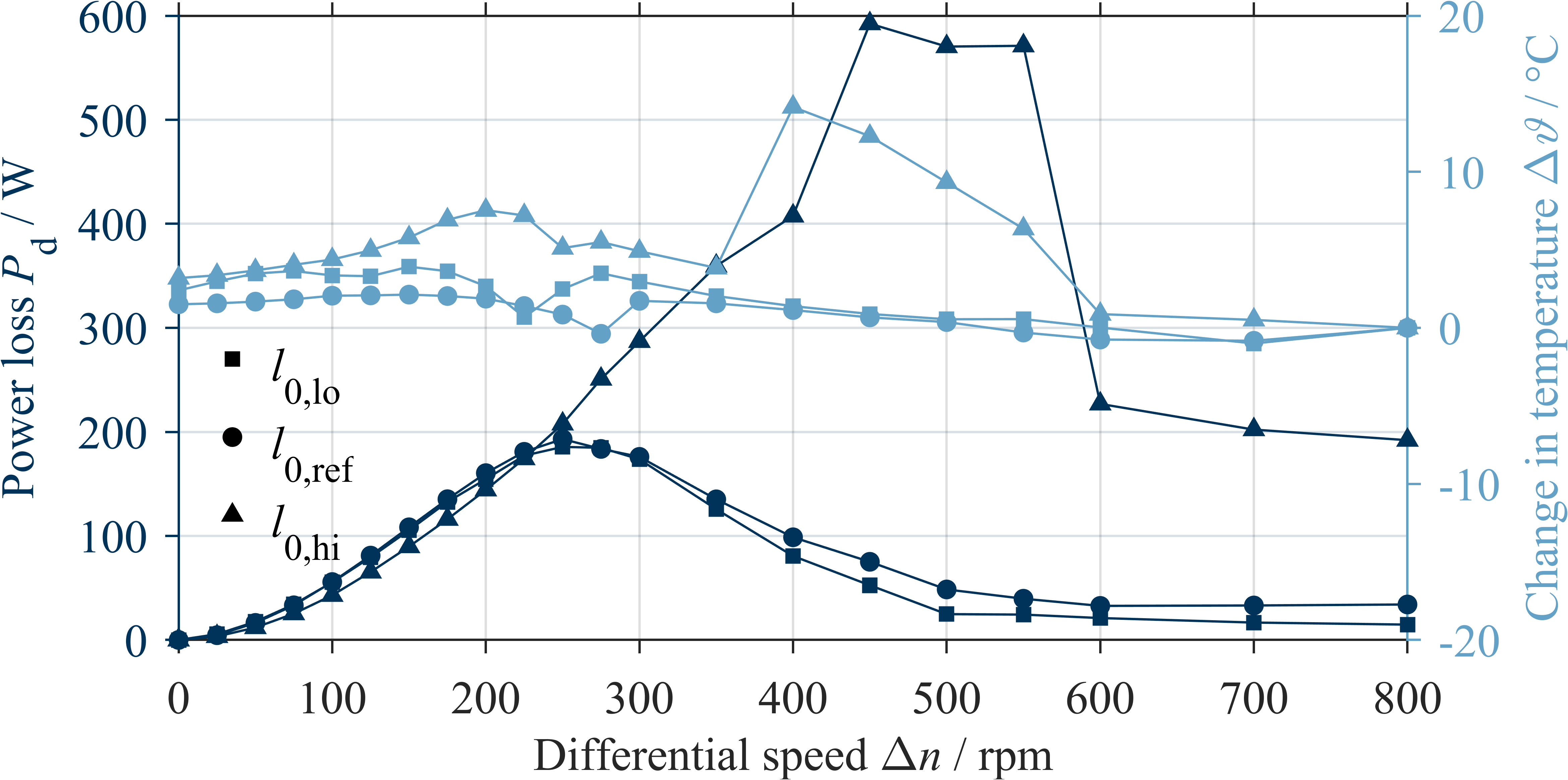


Supplementary Figure 2: Power loss and change in sump temperature.

**Video recordings**

Supplementary Video 1: Video recordings of the flow at specific differential speeds for the initial oil level *l*_0,ref_.

Supplementary Video 2: Close-up video recordings of the flow at specific differential speeds for the initial oil level *l*_0,ref_.

Supplementary Video 3: Video recordings of the flow at specific differential speeds for the initial oil level *l*_0,lo_.

Supplementary Video 4: Video recordings of the flow at specific differential speeds for the initial oil level *l*_0,hi_.

Supplementary Video 5: Video recording of the flow development when accelerating with 25 rpm/s.
